# Host Cell Proteases Involved in Human Respiratory Viral Infections and Their Inhibitors: A Review

**DOI:** 10.3390/v16060984

**Published:** 2024-06-19

**Authors:** Bailey Lubinski, Gary R. Whittaker

**Affiliations:** 1 Department of Microbiology & Immunology, College of Veterinary Medicine, Cornell University, Ithaca, NY 14850, USA; bl744@cornell.edu; 2 Department of Microbiology & Immunology and Public & Ecosystem Health, College of Veterinary Medicine, Cornell University, Ithaca, NY 14850, USA

**Keywords:** proteases, viruses, respiratory proteases, protease inhibitors, SARS-CoV-2

## Abstract

Viral tropism is most commonly linked to receptor use, but host cell protease use can be a notable factor in susceptibility to infection. Here we review the use of host cell proteases by human viruses, focusing on those with primarily respiratory tropism, particularly SARS-CoV-2. We first describe the various classes of proteases present in the respiratory tract, as well as elsewhere in the body, and incorporate the targeting of these proteases as therapeutic drugs for use in humans. Host cell proteases are also linked to the systemic spread of viruses and play important roles outside of the respiratory tract; therefore, we address how proteases affect viruses across the spectrum of infections that can occur in humans, intending to understand the extrapulmonary spread of SARS-CoV-2.

## 1. Introduction

Among the hurdles that viruses must overcome is the inability to actively adapt to new environments. One way to overcome this hurdle is to utilize host components to modify and activate viral proteins. The human body contains hundreds of proteases, enzymes that process the peptide bonds of both proproteins and proteins through hydrolytic cleavage. Proteases are essential for countless basic human life processes such as hormone and neurotransmitter activation and release. Likewise, proteolytic cleavage can be important for several steps in the life cycles of viruses, such as host cell entry, uncoating, and virion formation. While many viruses encode their own proteases—which are excellent drug targets—the ability to co-opt host cell proteases can be a major advantage, but one that has received comparably little attention for drug development. For respiratory viruses, the proteases present in the respiratory tract can be used for various viral functions, which can impact pathogenesis and cell tropism. As host proteases do not undergo the same selective evolutionary pressure as viral proteases, inhibitors that target host proteases may elicit a slower viral evolutionary response towards developing drug resistance. Identifying potent inhibitors of respiratory viruses has long been a goal for researchers and has become more of a priority with the advent of the COVID-19 pandemic. SARS-CoV-2, the causative agent of COVID-19, uses several proteases that were previously well known to be involved in the life cycles of other respiratory viruses, as well as illuminating new factors for viral infection. This has allowed researchers to explore foundational research on those viruses and use it as a guide for current drug development. This review will primarily focus on the proteases implicated in SARS-CoV-2 infection, the inhibitors used to disrupt that infection, and the background of these proteases and protease inhibitors in other respiratory viruses. 

## 2. Respiratory Proteases

**Figure 1:**
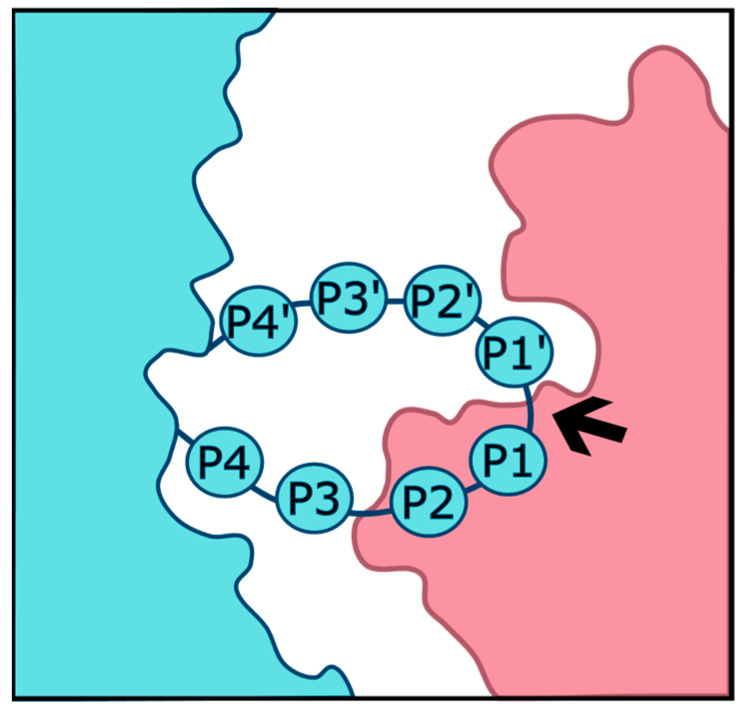
Substrate (**left**) cleaved by protease (**right**) with the scissile bond marked by an arrow.

Proteases recognize cleavage substrates through a variety of physical properties. The requirements that a substrate must meet in order to be cleaved vary by the protease of interest. Some proteases, for example, digestive enzymes such as trypsin, are quite general in their requirements and, thus, retain the ability to cleave a broad array of proteins. Others such as furin are much more specific. Among the properties that determine cleavability are the steric availability of the cleavage site to the protease, as well as the charge, polarity, and glycosylation status of the amino acids within and surrounding the cleavage site. Substrate cleavage sites are typically described using a numbering system introduced by Schechter and Berger [1]. The amino acids on the N terminal side of the cleavage site are numbered P1, P2, etc., with the numbering increasing as distance from the scissile bond increases. On the C terminal side, amino acids are similarly numbered, but as P1′, P2′, etc., as depicted in Figure 1. 

Respiratory proteases conduct a variety of important roles in the respiratory tract. They are involved in ciliary function, ion transport, mucus expression, and the immune response, and their dysregulation is tied to multiple disease states [2]. Proteases are categorized by their catalytic residue, and there are four main types of proteases found in the respiratory tract: serine, cysteine, metallo-, and aspartyl proteases. An excellent review of host cell proteases and their role in the cell’s immune response to respiratory virus infections has previously been published [3]. In this review, we shall focus on serine, cysteine, and metalloproteases and their well-characterized roles in facilitating the entry into the cell and the replication of respiratory viruses. 

Proteases from all three of these categories are co-opted by SARS-CoV-2, and their presence in the host has important implications for both transmission of the virus and pathology. The expression of ACE2 and TMPRSS2 that is highly homologous to human ACE2 and TMPRSS2 is known to be an important susceptibility factor in animal infection with SARS-CoV-2 [4,5,6,7,8,9,10]. Additionally, many of the human proteases are polymorphic. Genetic variants of furin, TMPRSS2, and ACE2 are all present in the human population at significant levels, and several studies have found that these variants have an impact on COVID-19 presentation [11,12,13,14,15]. This highlights that continued study of host proteases is essential for a more comprehensive understanding of viral infections and the factors that increase the potential for zoonotic spillover events. 

## 3. Serine Proteases

Serine proteases catalyze the cleavage of proteins via a nucleophile on a serine amino acid in the catalytic site of the enzyme. This serine can be a part of a catalytic triad or dyad. There are 50 different serine protease classes, further divided into 14 clans [16]. A wide variety of serine proteases have been found to proteolytically cleave the glycoproteins of respiratory viruses. Table 1 depicts selected well-studied proteases, their cleavage preferences, and the viruses with which they are associated. Serine proteases are the most common class of protease present in the lungs and, thus, are often utilized by viruses. 

### 3.1. Furin

Furin is a proprotein convertase that minimally cleaves at an R-X-X-R amino acid motif, a polybasic region that must be within an accessible portion of a protein. Furin is present in both the trans-Golgi network and endosomes, and it is essential for the processing of many host substrates crucial to homeostasis and the immune response. While the canonically recognized cleavage site is R-X-X-R, the “furin cleavage site”, or FCS, is actually much more complex, with furin interacting with its cleavage substrate at 30 amino acids spanning the cleavage site, with some amino acids interacting more strongly than others. A study looking at the interaction of furin with 130 known furin cleavage sites found that the properties of 20 of these amino acids were essential determinants of furin’s ability to cleave [53]. The canonical R-X-X-R motif lies within an eight-amino-acid-long core region of this larger motif. Within the core region, positively charged residues and flexibility are requirements, with specific amino acids needed at certain positions. Outside of the core region, the polarity of amino acids and accessibility to solvents are also determining factors of cleavage. Recent work on MHV has shown that what may appear to be minor changes in the FCS can have a pronounced effect [54]. In addition to specific amino acid side-chain requirements, it has been found that the glycosylation patterns of proteins (both N- and O-linked) can sometimes abrogate furin cleavage [55]. Furin cleavage requires a pH of 6–8.5 and the presence of Ca2+ in order to be able to cleave [56]. 

Furin cleavage of influenza hemagglutinin (HA) has been extensively studied as a pathogenicity determinant in the context of highly pathogenic avian influenza [57]. The HA cleavage site is essential for influenza infectivity as, when cleaved, the N-terminus of the fusion peptide is exposed. However, the cleavage of HA exhibits a certain amount of variability. Individual strains of influenza (low pathogenicity or high pathogenicity) may carry either a monobasic or polybasic cleavage site, which determines whether or not their HA can undergo furin cleavage or needs to be cleaved by another protease, such as HAT, TMPRSS2, and other trypsin-like proteases. The presence of a monobasic or polybasic cleavage site is the defining characteristic that separates low-pathogenic viruses from highly pathogenic viruses [58]. Strains of influenza with a monobasic cleavage site are reliant on the presence of trypsin-like proteases on the surface of cells during cell entry. These proteases are only expressed in certain cells; therefore, infection is restricted to those cells. Strains with a polybasic cleavage site are able to utilize furin as virions are being made; therefore, HA is already fusion-competent upon its release and the virions are able to infect cells that do not express trypsin-like proteases. While furin is a ubiquitous protease, it should be noted, however, that cells often express very low amounts of furin, and pathogenesis may be linked to expression levels in different tissues and cell types, e.g., endothelial cells [59]. Also, while other animal species do have furin homologs, there may be differences in the levels and localization of expression that could lead to alternate effects of polybasic cleavage sites on pathogenicity in those animals. However, in both ferrets and poultry, the ability of furin to process HA has been shown to be a determinant of the pathogenicity of highly pathogenic avian influenza (HPAI) [60,61]. 

**Figure 2:**
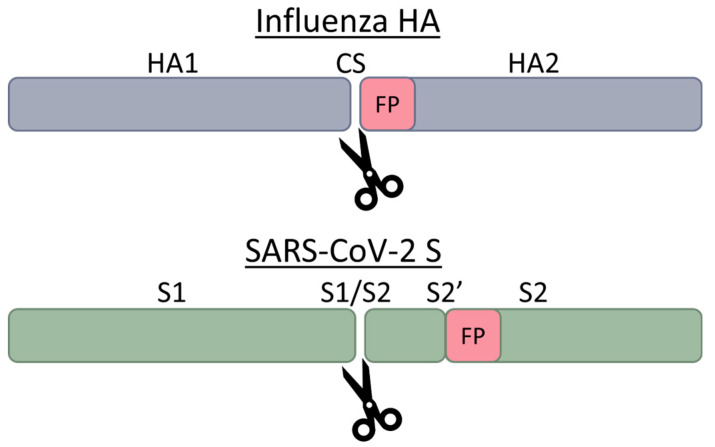
Furin cleavage of influenza HA and SARS-CoV-2 S. HA has one cleavage site (CS) while S has two (S1/S2 and S2′). The location of the fusion peptide of each glycoprotein is indicated by FP.

Viral envelope proteins are typically cleaved at a single position, but in some cases, there are two distinct cleavage sites (see examples in Figure 2). This was first noted for SARS-CoV-1 and then with other coronaviruses. Depending on the individual virus, furin cleavage may be considered a priming event, before the activation protease subsequently cleaves adjacent to the fusion peptide, as with influenza HA, HIV-1 Env, and for paramyxo- and pneumoviruses—this activation is typically through non-furin proteases (see below). Respiratory syncytial virus (RSV, or human orthopneumovirus) is distinct in having two furin cleavage sites, a property shared with MERS-CoV, although the proteases used may be cell-type specific [25,62]. 

**Figure 3:**
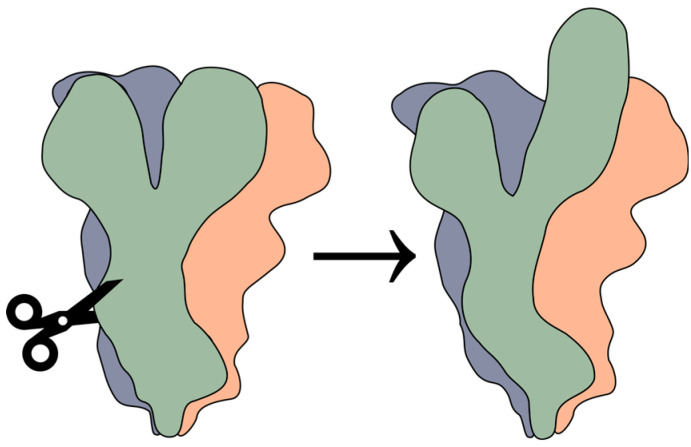
Artistic representation of one monomeric unit of SARS-CoV-2 S being cleaved by furin and then entering into the “up” conformation with the receptor binding domain exposed.

For SARS-CoV-2, furin cleavage of the spike (S) protein is important for the transmission and pathogenesis of the virus, the formation of syncytia, and the available entry pathway into the cell [63]. Furin processing allows for S to enter more easily into an “up” conformation that promotes receptor binding and acts as a “priming” event during virion formation (see Figure 3) [64]. This priming is essential for further proteolytic processing by either TMPRSS2 or other proteases. However, in the absence of this priming event, virion glycoproteins can instead be cleaved by cathepsins following uptake into the late endosome, leading to fusion with the endosomal membrane, as will be discussed further below. This entry pathway takes more time but allows for the virus to enter a wider range of cells. However, the furin/TMPRSS2 pathway has an additional benefit in that it allows for SARS-CoV-2 virions to evade elements of the immune system such as the interferon-induced transmembrane proteins present in the endosome [24]. Additionally, furin is known to be important for the formation of syncytia during SARS-CoV-2 infection. Syncytia are formed when S is trafficked to the host cell membrane during the course of infection. S then can fuse the cell membrane with the membrane of a neighboring cell, a process which is hypothesized to allow for cell-to-cell transmission of the virus and the evasion of the immune system [65]. 

As SARS-CoV-2 continued to spread and evolve in the human population, the mutations that accumulated around the FCS underscored the difference between evolutionary pressures in vivo and in vitro. While the virus readily loses the FCS via deletions and point mutations in vitro, the FCS has been strongly conserved in vivo [66]. Though several globally dominating variants have emerged with mutations near the FCS, these mutations have never completely interfered with furin processing. Rather, these mutations seem to take on the role of regulating the level of furin cleavage that occurs (P681H), with some mutations increasing the level of cleavage (P681R), as well as these point mutations having the ability to alter O-glycosylation of the FCS [67,68,69,70,71]. While S entering the “up conformation” is advantageous in many ways, it does make the S more susceptible to immune recognition as well as making it less stable. This stability decrease has been lessened by other mutations in S such as D614G that may allow the S to undergo a higher level of cleavage with fewer disadvantages [72]. Modification of the FCS via point mutations also occurred extensively as different isolates of the prototype coronavirus MHV were isolated, and in certain other coronaviruses, (OC43) modulation of the FCS may occur through an indel, in addition to point mutations [73,74]. Thus, modulation of furin cleavage through natural selection is a powerful means of regulating CoV tropism and pathogenesis; the virus continues to need to balance the efficiency of the transmission benefit of furin cleavage with the need to evade the immune response, now that most people have some level of pre-existing immunity. 

For coronaviruses, furin-mediated cleavage (via priming at S1/S2) is linked to fusion activation at S2′, but it may also serve a more general role in virion maturation. In this case, furin may be playing an analogous role to that of other viral glycoproteins such as HIV-1 Env and filovirus GP. For the filoviruses EBOV and MARV, furin cleavage precedes the critical cathepsin-mediated cleavage events needed for virus entry, and it is interesting to note that the FCS is found in a quite different position in the GP protein sequence, implying a distinct maturation process between EBOV and MARV [75]. A similar role for furin is found for the DENV prM protein, where it controls the proportion of mature vs immature particles that form, which in turn may affect ADE and viral pathogenesis [76,77,78]. prM that is not cleaved by furin during virion formation can still be cleaved in the endosome during ADE by cathepsins. This alternative cathepsin cleavage route also can take over when SARS-CoV-2 S is not cleaved by furin, leading to furin cleavage being important for infection but not essential. 

### 3.2. Coagulation Cascade Proteases (Plasmin, Thrombin, Factor Xa) 

It is important to keep in mind that the host cell components that viruses take advantage of have important roles in maintaining homeostasis. The Blood Coagulation Cascade, which relies on several different proteases, is essential to stop blood loss following injury. Among the contributors to the cascade are thrombin, Factor Xa, and plasmin. These proteases can be responsible for cleaving other proteins within the pathway, or they can activate various cell types to respond to injury via the cleavage of Protease Activated Receptors (PARs), which are ubiquitously expressed [79]. This cascade exists in a carefully regulated balance, and viral disruption of this balance in either direction can lead to pathology. Respiratory viral infections are known to increase the risk of deep vein thrombosis and pulmonary embolism, as well as other serious vascular complications [80]. This can be caused by either the upregulation of coagulant factors or the downregulation of anticoagulant proteins. Several respiratory viruses can utilize the coagulation proteases for glycoprotein cleavage. This has been posited to form a positive feedback loop, where the glycoprotein is cleaved, leading to higher cell entry, which leads to the immune response of increased production of coagulation factors, which in turn leads to increased glycoprotein cleavage [21]. 

While influenza virus infection in the human respiratory tract is now known to be driven by trypsin-like proteases, the initial breakthrough for our understanding identified plasmin as the key activating protease [81]. The virus strain studied was the neuro-adapted isolate WSN, which had adapted to use plasmin during passage to make it trypsin-independent for growth in cell culture and in the murine brain [82]. Some clinical strains of influenza are also able to utilize plasmin for HA activation [42]. Early studies on SARS-CoV-1 showed a role for plasmin-activated cleavage, primarily at S2′ (R797), along with trypsin and TMPRSS11a; for SARS-CoV-2, plasmin has been indicated as one of the many proteases capable of activating spike [21,44]. Notably, RSV also is activated by plasmin, along with trypsin and thrombin [43].

Influenza HA activation, which is normally dependent on trypsin-like proteases or furin, is thought to lead to dependence on Factor Xa when the virus is adapted to grow in eggs, which is still a common method of growing influenza stocks (e.g., vaccine seeds) [83]. In contrast, SARS-CoV-2 does not appear to need adaptation to allow the use of a range of systemic secreted proteases such as plasmin, thrombin, and Factor Xa [21]. However, the impact of these proteases during an in vivo infection has yet to be fully elucidated. 

### 3.3. Elastase

In vivo, elastase is produced mainly in the pancreas but also exists in a neutrophil-secreted form as part of the host’s innate immune defenses. The use of elastase as an activating protease has been reported for SARS-CoV-1, which may play a factor in promoting the initial inflammatory response to infection, but the situation for SARS-CoV-2 is less clear [36]. As with any virus-activating protease, the enzyme exists in tissue as part of a tightly regulated system, with neutrophil elastase proposed to be involved in ACE2 shedding, and so it has a protective effect for COVID [84]. Interestingly, engineering influenza HA to be elastase-cleaved has been used as a means to generate a live-attenuated vaccine virus [85]. Elastase-dependent influenza is attenuated, as elastase is not present in high enough levels during infection to make up for the lack of TMPRSS2 or furin cleavage during virion formation. This highlights the fact that it is not enough to have a proteolytic cleavage site on the glycoprotein; the cleavage site needs to be able to be cleaved by specific proteases that are present in specific locations during infection.

### 3.4. Type II Transmembrane Serine Proteases (TTSPs)

Type II Transmembrane Serine Proteases (TTSPs) all have a serine protease domain of chymotrypsin with a catalytic triad of histidine, aspartic acid, and serine [86]. Their catalytic triad leads to these proteases being considered trypsin-like. TTSPs are attractive proteases for viruses due to their location on the surface of epithelial cells, allowing for the possibility of glycoprotein processing during cell entry. There are at least nine TTSPs that are found in the respiratory tract, and they are often involved in viral glycoprotein processing. Low pathogenic strains of influenza are known to rely on TTSPs for glycoprotein activation. Table 2 depicts select TTSPs that are known to be utilized by respiratory viruses. 

### 3.5. TMPRSS2

TMPRSS2, or Transmembrane Serine Protease 2, is a member of the hepsin/TMPRSS subfamily of TTSPS, which consists of at least seven similar proteases [86]. It cleaves at sterically available monobasic sites, where an arginine or lysine is present. The role of TMRPSS2 has yet to be fully elucidated, with it having proposed roles in fertility, inflammation moderation, prostate protection, and tumor suppression [107]. TMPRSS2 is present throughout the respiratory tract in the lung, bronchus, larynx, trachea, vocal folds, buccal mucosa, nasal mucosa, and tonsils [108]. The level of TMPRSS2 expression is tightly regulated in each tissue type, with over-expression of TMPRSS2 being associated with pathological states such as cancer. Inhibition of TMPRSS2 occurs naturally through host-made factors such as plasminogen inhibitor 1 and hepatocyte growth factor activator inhibitor (HAI-2). However, TMPRSS2 is also associated with the coagulation cascade proteases discussed above. TMPRRS2, trypsin, matriptase, and Factor Xa are all able to activate PAR2, which leads to the increased expression of matrix metalloproteases. These matrix metalloproteases are also known to be involved in viral life cycles; thus, even when TMPRSS2 does not have a direct interaction with the virus, it may still affect the outcome of infection through other mechanisms. 

Its involvement in many respiratory virus life cycles can be readily explained by its high level of expression throughout the respiratory tract. TMPRSS2 is involved in the direct proteolytic activation of many viral glycoproteins and was first identified as a viral cofactor for influenza [91]. TMPRSS2 processes the influenza HA during virion formation, an essential step that renders HA fusion competent. TMPRSS2 also acts as a proteolytic activator during the cell entry of several coronaviruses, including SARS-CoV-1, SARS-CoV-2, HCoV-229E, and MERS-CoV. The activation of these viral glycoproteins by TMPRSS2 is an essential step for entry at the cell surface as it allows these glycoproteins to change into their fusion-competent conformations. TMPRSS2′s cleavage specificity is also broad enough that it is able to cleave cleavage sites that are traditionally considered to be “furin cleavage sites”. TMPRSS2 is interestingly also involved in the cleavage of ACE2, an additional host cell protease that will be discussed in depth later in this paper. This processing of ACE2 has been found to augment SARS-CoV-1’s cell entry, in addition to TMPRSS2’s processing of the SARS-CoV-1 S [109]. Interestingly, while the S1/S2 cleavage site of SARS-CoV-2 has mutated throughout the course of the COVID-19 pandemic, the S2′ site, which TMPRSS2 is able to cleave, has remained unaltered [110,111]. 

## 4. Cysteine Proteases–Cathepsins

Cathepsins are a type of cysteine protease that resemble papain, leading to them also being referred to as papain-like proteases. Their active site consists of a catalytic dyad with a histidine residue and a cysteine amino acid acting as the nucleophile [112]. Cathepsins are generally endosomal proteases but can also be found in other locations. For example, cathepsin L is present in endosomes and lysosomes but is also secreted outside of the cell. The level of cathepsin L in the blood is correlated to the level of pathogenesis following viral infection and is also elevated during other noninfectious pathological states [113]. Cathepsins need to be exposed to an acidic environment to be activated, and they prefer acidic and reducing conditions for cleavage. These proteases participate in a variety of virus life cycles and often are integral for alternative entry pathways. Cathepsins cleave viral glycoproteins in these so-called alternative pathways for several respiratory viruses, along with coronaviruses such as SARS-CoV-1, SARS-CoV-2, and MERS-CoV. For SARS-CoV-2, later variants show a preference for the cathepsin pathway in cell culture, despite this shift not occurring in vivo or in human airway organoids [114,115,116]. Beyond just glycoprotein cleavage, cathepsins also are used by viruses to modulate the immune response through several different pathways [117].

As a part of the Mammalian Orthoreovirus (MRV) life cycle, the virions’ capsids must be proteolytically digested away to become Infectious Subvirion Particles (ISVPs), which are then able to penetrate the endosomal membrane and gain entry into the cell [118]. This proteolytic digestion of the capsid has been attributed to several different cathepsins, making cysteine proteases essential for MRV infection. Specific point mutations in the MRV protein that undergo cleavage have been found to increase the efficiency of cathepsin cleavage [119,120]. However, despite proteolytic cleavage being considered the rate-limiting step of infection, few strains of MRV have incorporated these mutations, and viruses with these mutations lose titer more rapidly at elevated temperatures when compared to wild-type virus [121]. This reaffirms that proteolytic cleavage requires a careful balance between cleavage efficiency and overall viral fitness. Cathepsin cleavage is intimately tied to the route of entry for reovirus, and has been reviewed in Mainou and Dermody 2012 [122].

Many other virus systems take advantage of cathepsin cleavage in endosomes. Perhaps the most notable of these is the Ebola virus, whereby, following non-specific binding at the cell surface and internalization, cathepsin L and cathepsin B degrade the filovirus GP down to an 18kDa “stub”, which is the fusion-active component upon engagement with the NPC-1 receptor [123]. Another not classically respiratory virus (albeit transmitted via the oro-nasal route) is Nipah virus; in this case, the F protein enters recycling endosomes where it encounters cathepsins as part of the virus assembly pathway [124,125]. Cathepsins are also involved in RSV infection, via cleavage of the G (attachment) protein, and influenza infection via unspecified means [126]. Table 3 lists 6 different cathepsins, along with their cleavage determinants and the respiratory viruses that use them. 

## 5. Metalloproteases

Metalloproteases differ from serine and cysteine proteases in that instead of using an amino acid as a nucleophile in their hydrolytic reaction, metalloproteases use a water molecule that has been activated by a metallic cation. Some metalloproteases have been found to cleave viral glycoproteins, such as ADAM and MT-MMP. However, this function is less well studied from the perspective of viruses than other groups of proteases.

Many metalloproteases also impact the viral life cycle in less direct ways. Matrix metalloproteases (MMPs) in particular are important players in many pathways that regulate the immune response and wound repair. MMPS are regulated by Tissue Inhibitors of Metalloproteases (TIMPs), and the interruption of this careful balance between activity and inhibition is associated with many disease states [142]. RSV is known to induce the expression of many MMPs. Drugs targeting these proteases were found to enhance viral clearance, inhibit syncytia formation, and prevent viral replication [52,143]. MMP-9 specifically is also implicated in infection with other respiratory viruses, such as influenza, rhinovirus, and human parainfluenza virus [144,145,146]. The exact mechanism of action of these MMPs in enhancing viral infection is unknown and remains mainly as hypotheses, based on either MMPs’ role in restructuring the respiratory tract or their role in the immune response. 

MMPs were directly shown to act on the coronavirus spike, first through a study of MHV where MMP and ADAM family proteases acted in a strain-dependent manner, and they have more recently been added to the list of activating proteases for SARS-CoV-2 [147,148,149]. However, COVID-19 infections are also impacted by the role that MMPs play in the immune system. SARS-CoV-2 infections are known to increase the level of MMPs, which can lead to tissue damage and contribute to a cytokine storm reaction [150,151,152,153,154]. The role of MMPs is complex and involves multiple biological pathways. An excellent review of MMPs and COVID-19, as well as other coronavirus infections, has been published by Salomão et al. [155].

### ACE2

Another metalloprotease of interest is Angiotensin-Converting Enzyme II (ACE2), which, as the name suggests, regulates angiotensin II levels through hydrolytic cleavage. Its position at the cell surface allows it to function as a receptor for SARS-CoV-2. The S1 subunit of the spike protein binds to ACE2, which begins the process of the cell being infected [156]. This action does not directly cleave the spike protein, but it does allow for the spike protein to be cleaved by other proteases, such as the previously discussed TMPRSS2 [157]. This use of proteases as receptors, and not for their proteolytic capacity, is also seen in several other coronaviruses, and the pharmacological intervention in this receptor binding interaction is a major area of research. Drugs targeting the SARS-CoV-2 spike and ACE2 are not focused on blocking the proteolytic activity of ACE2. Rather, research focuses on ACE2 decoys that will bind the spike protein before the virus arrives at a cell, thereby preventing successful receptor binding and infection [158]. Recently ACE2 was also revealed to act as a regulator of TMPRSS2 activity. This regulation occurs through a non-catalytic mechanism but has been shown to increase TMPRSS2 activation of influenza A and MERS-CoV [159]. When we think of the roles of host proteases in viral infection, it is tempting to think of them as having a straightforward interaction with viral glycoproteins, i.e., ACE2 acts as a receptor for SARS-CoV-2 S. However, we must remember that these proteases exist in a complex and ever-changing environment, and they have important roles that they serve within the host. The relationship between proteases and viral infections cannot be simple, because the system that this relationship exists in is not simple. Table 4 lists several metalloproteases utilized by respiratory viruses, and illustrates some of the different roles that these proteases can fulfill in the viral life cycle. 

## 6. Protease Inhibitors

**Figure 4:**
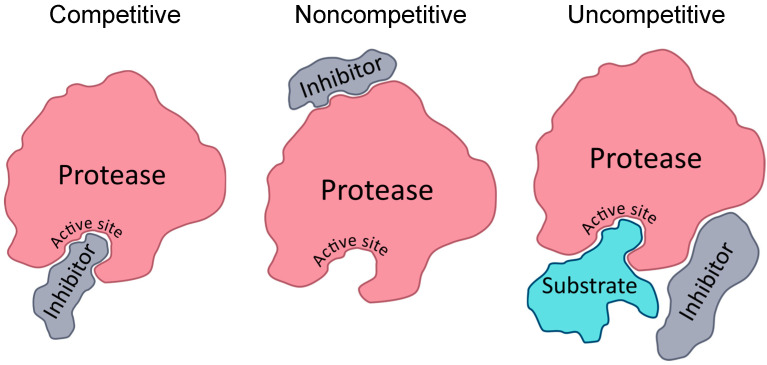
Illustration of the different types of inhibition.

Protease inhibitors, like proteases themselves, are a broad and diverse group. Inhibitors play an essential role in maintaining homeostasis, preventing proteases from being overly active. Due to this role, protease inhibitors have been found in many life forms, leading to a large group of existing inhibitors to explore as drug candidates. Inhibitors can be classified by the type of protease they inhibit or, like other enzyme inhibitors, by their mode of action. When an inhibitor binds to the enzyme through a covalent interaction, this is considered an irreversible reaction. These inhibitors fall into either the less specific group of affinity-labeling inhibitors or the more specific suicide inhibitors, which require the protease to enzymatically act upon them to achieve inhibition [164]. For reversible inhibitors, inhibitors noncovalently interact with the protease, and the location of that interaction determines whether it is competitive, noncompetitive, or uncompetitive inhibition. Competitive inhibitors occupy the active site of the protease, noncompetitive inhibitors bind elsewhere on the protein, and uncompetitive inhibitors only bind to enzymes that are already bound to their substrates (see Figure 4). Inhibitors can be proteins, peptides, small molecules, or antibodies. They can directly interact with the protease, or they can interact with molecules that proteases need to be active, i.e., Ca2+ chelators that inhibit furin.

Because the kinetics of inhibition differ between the inhibitor types, whether an inhibitor is reversible or irreversible is a determinant of its appeal as an antiviral. Irreversible inhibitors are attractive because of how long they are active and the ease with which they can be designed, but they have more potential for negative side effects, especially if they are widely active on many proteases [165]. Both the length of activity and the potential for negative side effects stem from the fact that the inhibition is irreversible. Once a protease is inhibited, a cell will need to make new protease in order to regain proteolytic function. An irreversible inhibitor that is broadly acting could disrupt many processes involved in homeostasis. Therefore, irreversible inhibitors have a higher requirement for specificity and subcellular targeting design than reversible inhibitors. Competitive reversible inhibitors are also more straightforward to design, due to their need to fit into the active site of the enzyme, but they require a high concentration to compete with other substrates. This can be a barrier when trying to inhibit the cleavage of viral proteins in subcellular compartments, as you need to get a large quantity of the inhibitor into those specific compartments. Uncompetitive and noncompetitive reversible inhibitors do not have this same issue with concentration; however, these types of inhibitors are complicated to design. However, the problems of irreversible and competitive inhibitors both have the same solution, cellular and subcellular drug targeting. Directing inhibitors to the correct location would allow the concentration to reach a high level in the area of interest without the need for an extreme dose to the rest of the body. Ideally, the inhibitor would remain inert until it reached its target destination, lowering the chances of adverse side effects. Research is quickly advancing, both in drug modifications that direct drugs to be taken up into specific cell compartments, as well as in the creation of nanoparticle carriers that allow for precise drug targeting, as well as having other benefits [166,167].

The human genome encodes for numerous protease inhibitors, as maintaining a careful balance of proteolytic activity is essential for homeostasis. However, these endogenous inhibitors also carry out important roles in the immune response to viral infections. The viruses that are inhibited by endogenous inhibitors are diverse and include coronaviruses, flaviviruses, and filoviruses, among others [168]. This inhibition can be a direct interruption of the catalytic mechanism of cleavage, a rerouting of proteases to an alternate cellular compartment, or an interaction with the protease in one of its other functional domains. A comprehensive review of these endogenous inhibitors has been carried out by Lotke, Petersen, and Sauter [168]. Of particular interest among these inhibitors are serpins. 

Serine protease inhibitors (serpins) are present in most types of life. These inhibitors mostly inhibit serine proteases but select serpins can also inhibit other types of proteases. Serpins contain cleavage motifs of the proteases that they inhibit, and upon their cleavage, they undergo a conformational change that covalently traps the protease in an irreversible interaction [169]. This conformational change is irreversible; thus, they are suicide inhibitors. Interestingly, poxviruses, along with some plant viruses and herpesviruses, have been found to encode their own serpins. Poxvirus serpins are the most highly studied and have been found to exhibit anti-apoptotic and anti-inflammatory roles that can promote viral replication [170]. Serp-1, a serpin encoded in the myxoma virus (MyxV) genome, has been tested as a therapeutic target for several inflammatory disorders as well as viral infections with notable success, as outlined in the review by Varkoly et al. [171]. By inhibiting the urokinase-type plasminogen activator receptor, complement receptors, and some of the thrombolytic enzymes (such as the previously discussed plasmin), it can prevent the overactivation of the immune response [172,173,174]. The success of using a viral serpin, which was originally made to support a viral infection, to then treat disease caused by viral infections is highly promising and supports the notion that the viral use of host proteases exists in a balance which we can seek to disrupt. 

## 7. Current Therapeutic Strategies Targeting Coronaviruses, Influenza Viruses, and Para-Myxoviruses

The COVID-19 pandemic prompted a resurgence of therapeutic development for respiratory viruses, and while some drugs targeting SARS-CoV-2 are now approved for use in humans, many additional drugs are currently approved in development, including inhibitors of the viral polymerase and viral Mpro and 3CL-like proteases [175]. Recent oral inhibitors now available include Paxlovid^®^, and while this remains in use, Molnupiravir^®^ and the injectable Remdesivir^®^ are no longer widely recommended, and most therapeutic monoclonal antibodies have been discontinued. Currently, the main therapeutics targeting influenza are the DAAs Tamiflu^®^ and Relenza^®^ (targeting the viral neuraminidase), with Baloxavir^®^ also now available (targeting the viral polymerase). However, numerous influenza virus strains are emerging that are resistant to Tamiflu^®^ and Relenza^®^, rendering the treatment ineffective. For paramyxoviruses and pneumoviruses, ribavirin has been utilized for RSV treatment and shows similar in vitro neutralization of HMPV [176], but there are significant questions concerning its efficacy in clinical settings. Based on successes with RSV, humanized monoclonal antibodies, the primary prophylactic treatment for HRSV, are currently in development for HMPV, but the cost of such treatment is extremely high. Host-targeted approaches are also in development and have the notable potential benefit of limiting the emergence of antiviral-resistant viruses.

- Inhibition of furin as an antiviral strategy

Given its importance for viral infection, furin has received notable attention as a therapeutic option [177]. The first generation of furin inhibitors were peptide-based and lacked specificity, and were followed by serpins such as AT-PDX, which have high specificity and increased potential as therapeutic agents. Recent studies with bioavailable small molecule furin inhibitors such as BOS-981 that effectively inhibit SARS-CoV-2 show promise as candidate therapeutics, especially when combined with TTSP inhibitors (see below) [178]. Ca2+ chelators, serpins, and proprotein convertase inhibitors have all been successful at inhibiting furin. Most known inhibitors of furin have inhibitory effects on other proteases as well, so the development of new, targeted furin inhibitors is still an area of intense interest. However, as some viruses are able to utilize multiple proteases for glycoprotein cleavage, it is important to design treatments that carefully balance the need to prevent off-target effects while still targeting the diverse set of proteases that viruses utilize.

- Inhibition of TTSPs as an antiviral strategy

Inhibition of TTSPs is among the most promising alternative host-targeted approaches under consideration as therapeutics or prophylactics, for SARS-CoV-2 or other respiratory pathogens. The use of TTSP inhibitors was originally investigated for the treatment of influenza, as an alternative to inhibitors that target the influenza protease neuraminidase [179,180,181]. Camostat, a TTSP inhibitor repurposed as an antiviral, is currently in clinical trials for COVID-19—although currently with only limited success [182]. Notably, camostat has relatively low potency and is taken orally, which may contribute to its limited success in targeting respiratory disease. Impeding proteolytic activation of the viral HA via the serine protease inhibitor aprotinin [183], which has anti-fibrinolytic properties, has also been explored, including preclinical development and some limited clinical trials. The partial success of these trials reinforces the concept of targeting host cell-mediated proteolytic activation of viral glycoproteins as a viable therapeutic strategy. However, the development of aprotinin has limitations. First, being proteinaceous, indications are that aprotinin has a very short biological half-life, on the order of 0.7 h [184]. Second, it is currently unclear exactly which proteases are active in the human respiratory tract, and so which proteases need to be targeted. While aprotinin is somewhat broadly acting, it is likely to only target a subset of proteases. Importantly, it is a bovine-derived product, likely contributing to its toxicity and removal from the market [185]. Therefore, the development of alternative protease-targeted therapeutics in addition to aprotinin is likely to significantly expand the therapeutic options available to treat respiratory viruses. 

As proof of principle for this approach, a lead protease inhibitor comprising the Kunitz type, serine protease inhibitor SPINT2, also known as HAI-2, was tested [186]. SPINT2 is a 25 kDa protein found on the plasma membrane of a number of tissues, where it is mainly bound to the serine protease matriptase. Both matriptase and SPINT2 are initially located on the plasma membrane but are proteolytically released into the extracellular space. SPINT2 contains two Kunitz-type inhibitor domains that potently modulate the proteolytic activity of several trypsin-like serine proteases, including matriptase and hepatocyte growth factor activator; inhibition of these two proteases is thought to be the primary biological role of SPINT2. The properties of SPINT2, therefore, made it an excellent proof of principle to efficiently inhibit viral replication, including both influenza and HMPV [185,187]. Small molecule approaches previously discovered included an initial lead compound, IN-1 (N-0100), as an influenza therapeutic [94]. This was developed into N-0385, a small molecule peptidomimetic with a ketobenzothiazole warhead. In mice, N-0385 treatment reduced morbidity and mortality associated with influenza H1N1 and H3N2 infection, as well as proving highly effective in mice, to achieve progress towards the goal of prophylactic inhibition of a broad range of respiratory viruses [188].

## 8. Conclusions

The human genome encodes for hundreds of diverse proteolytic enzymes that are vital for the maintenance of homeostasis. These proteases are sometimes co-opted for virion maturation, cell entry, and immune modulation, among other things. These proteases provide an additional area to focus on in antiviral development. The COVID-19 pandemic has helped further the field of antiviral development but has also revealed complexities in the relationship between viruses and proteases that make designing antivirals more challenging. However, the field is readily rising to the challenge, and the field of antivirals targeting host cell proteases remains one of great interest. 

## Figures and Tables

**Table 1:** Characteristics of well-known serine proteases outside of the TTSP family.

Serine Proteases	Cleavage Preferences	Exploiting Respiratory Viruses
Chymase	Aromatic amino acids at P1, aliphatic amino acids from P2 to P4, S at P1′, E/D at P2′, A/V/G at P3′ [17]	Mammalian Orthoreovirus [18]
Factor Xa	Preference for PXG/AR↓XXD [19]	SARS-CoV-1 [20], SARS-CoV-2 [21]
Furin	R-X-X-R↓S [22]	Influenza virus [23], SARS-CoV-2 [24], MERS-CoV [25], RSV [26], HPIV [27], measles virus (MeV) [28], mumps virus [29]
Kallikrein-related peptidase 13 (KLK13]	V/Y-R/L/F/M-R↓ [30]	HKU-1 [31]
KLK1	R/Y↓S/R [32]	Influenza virus [33]
KLK5	X(aliphatic/aromatic)-R/K -X(polar/aliphatic)- R↓ [30]	Influenza virus [33]
Neutrophil elastase	A/V/I/T↓ [34]	Mammalian Orthoreovirus [35], SARS-CoV-1 [36,37]
PC5/6	R-X-R/K-R↓ [38]	Influenza virus [39], RSV [39]
Plasmin	R/L↓ [40], preference for aromatic hydrophobic residue at P2 [41]	Influenza virus [42], RSV [43], SARS-CoV-1 [44], SARS-CoV-2 [45]
Thrombin	L-X-P-R↓S/A/G/T-X(aromatic)-R [46]	RSV [43], SARS-CoV-2 [21]
Trypsin	K/R↓ [34]	SARS-CoV-1 [47,48], HMPV [49], RSV [43], HPIV [50]
Cathepsin G	Preference for F, Y, W, or L at P1 [51], S at P6, negatively charged amino acid in P2′ position	Mammalian Orthoreovirus [18], RSV [52]

**Table 2:** Characteristics of select Type II Transmembrane Serine Proteases.

Type II Transmembrane Serine Proteases (TTSPs)	Cleavage Preferences	Exploiting Respiratory Viruses
DESC1	R-R/A/L-L-A↓ [87]	Influenza virus [88], MERS-CoV [88], SARS-CoV-1 [88]
Human Airway Trypsin-like Protease	R/K↓ [89,90]	Influenza virus [91], HCoV-229E [92], SARS-CoV-1 [93], Mammalian Orthoreovirus [18]
Matriptase	Minimum: R/K [34]↓ Preferred: R-X(non-basic)-S-R↓ [87]	Influenza A virus [94,95]
TMPRSS11a	Unconfirmed, putative R/K↓	SARS-CoV-1 [44], influenza virus [96], MERS-CoV [96]
TMPRSS13/MSPL	R/K↓, preference for dibasic P2-P1 [97]	Influenza virus [88], SARS-CoV-1 [88], MERS-CoV [88], SARS-CoV-2 [98]
TMPRSS2	R/K↓ [99]	Influenza A + B virus [91], SARS-CoV-2 [100], HMPV [101], HCoV-229E [92], MERS-CoV [102], SARS-CoV [102], HPIV [103], Mammalian Orthoreovirus [18]
TMPRSS4	Unconfirmed, putative R/K↓ [104]	Influenza A virus [105], SARS-CoV-2 [106]

**Table 3:** Characteristics of select cathepsins.

Cysteine Proteases	Cleavage Determinants	Exploiting Respiratory Viruses
Cathepsin L	Prefers aromatic or aliphatic residues in P2 [127]	HCoV-229E [92], SARS-CoV-1 [128,129,130], SARS-CoV-2 [131], Mammalian Orthoreovirus [132], MERS-CoV [133], Nipah [134], RSV [126], Hendra [134]
Cathepsin B	Prefers an aromatic or aliphatic residue and tolerates a basic P2, an aromatic residue in P1′ and a P3′ G [127]	Influenza A virus [135], Mammalian Orthoreovirus [132], Nipah [124], SARS-CoV-2 [136]
Cathepsin S	Prefers aliphatic residues in P2, G/E in P1 [127]	Mammalian Orthoreovirus [137], SARS-CoV-2, SARS-CoV-1, RSV [52]
Cathepsin W	W/F–L/V–G/A/R↓V–D/N/E/Q (suggested [138])	Influenza A virus [139], RSV [52]
Cathepsin K	Prefers non-aromatic hydrophobic residues in P2 [140]	SARS-CoV-2 [136]
Cathepsin V	Prefers hydrophobic residues in P2, P in P3 [141]	SARS-CoV-2 [136]

**Table 4:** Characteristics of select metalloproteases.

Metalloprotease	Exploiting Respiratory Viruses	Role
MT-MMP	SARS-CoV-2 [149]	Proteolytic cleavage
ADAM	SARS-CoV-2 [149]	Proteolytic cleavage
ACE2	SARS-CoV-2 [160], SARS-CoV [161], HcoV NL63 [162]|Influenza A virus [159], MERS-CoV [159]	Receptor|Regulation of TMPRSS2
APN	HCoV-229E [163]	Receptor
